# The Significance of Sidedness in Patients with Metastatic Colorectal Cancer Treated with Triplet First-Line Chemotherapy

**DOI:** 10.31557/APJCP.2019.20.10.2891

**Published:** 2019

**Authors:** Shouki Bazarbashi, Ayman T Omar, Leen Raddaoui, Ahmed Badran, Ahmed Alzahrani, Ali Aljubran, Tusneem Elhassan

**Affiliations:** 1 *Section of Medical Oncology,*; 5 *Research Unit, Department of Oncology, King Faisal Specialist Hospital and Research Center,*; 3 *College of Medicine, Alfaisal University, Riyadh, Saudi Arabia,*; 2 *Department of Clinical Oncology and Nuclear Medicine, Suez Canal University Hospital, Ismailia,*; 4 *Department of Clinical Oncology, Faculty of Medicine, Ain Shams University, Cairo, Egypt. *

**Keywords:** Colorectal cancer, sidedness, chemotherapy

## Abstract

**Background:**

Recent data have shown that right-sided colon cancer carries poorer prognosis compared to left-sided tumors. This study was aimed to evaluate the progression-free survival, overall survival of patients with metastatic colon cancer of right-sided versus left-sided primaries treated with triplet chemotherapy regimen.

**Methods:**

The medical records of patients with metastatic colorectal cancer treated on phase I-II trial of combination Irinotecan, oxaliplatin, capecitabine, and bevacizumab were reviewed for sidedness of the primary. The analysis was performed for progression-free survival and overall survival according to the sidedness and other known prognostic factors.

**Results:**

Out of 53 patients treated with triplet therapy, 11 had right sided and 42 had left-sided primaries. The median age for right-sided primaries was 46 (range 24-55) compared to 53 (range 32-74) in left-sided primaries. Median progression-free survival was 14 months for right vs 18 months for left sided tumors (Hazard ratio 0.72, 95% confidence interval 0.27-1.88, p=0.492) and median overall survival was 21 months for right vs 29 months for left sided tumors (Hazard ratio was 0.86, 95% CI 0.32-2.26, p=0.752).

**Conclusion:**

First-line triplet chemotherapy may overcome the difference in prognosis between right sided and left sided primaries in metastatic colorectal cancer. A larger analysis is warranted.

## Introduction

Metastatic colorectal cancer carries a poor prognosis. Cure, for the most part, is only possible in a small subset of patients with resectable metastasis, and rarely with chemotherapy alone. Various clinical, pathological and molecular prognostic factors have been identified to influence the clinical outcome of metastatic colorectal cancer. This include but not limited to age, the organ involved, carcinoembryonic antigen, performance status at the time of metastasis, Neutrophil-Lymphocyte ratio, histological grade, RAS and BRAF mutation, microsatellite status and many more (O’Dwyer et al., 2001; Kohne et al., 2002; Popat et al., 2005; Tran et al., 2011; Mekenkamp et al., 2012; Rodriguez-Salas et al., 2017). Tumor location in right vs left sides of the colon has been identified as an independent prognostic factor for survival in metastatic disease (O’Dwyer et al., 2001). Two recent meta-analyses have clearly shown that right-sided colon cancer carries poorer prognosis compared to left-sided tumors, irrespective of the stage, race, adjuvant chemotherapy, number and quality of studies included (Yahagi et al., 2016; Petrelli et al., 2017). Recent retrospective data have suggested lack of benefit for right-sided metastatic colon cancer from the addition of anti-epidermal growth factor receptors anti-bodies to chemotherapy (Moretto et al., 2016). There is also smaller evidence from post-hoc analysis which showed that patient with right-sided metastatic disease benefit more from upfront triplet chemotherapy regimens than patients with left-sided tumors (Cremolini et al., 2015). We have previously published our results with the combination of capecitabine, oxaliplatin, irinotecan, and bevacizumab in the first-line setting of metastatic colorectal cancer within a phase I-II trial (Bazarbashi et al., 2015). In this analysis, we report the outcome of patients with right-sided versus left-sided primary tumors in patients treated on the above study and examine if triplet therapy does overcome the poor prognosis of right-sided metastatic colorectal cancer. 

## Materials and Methods

The medical records of patients treated with triplet chemotherapy consisting of irinotecan, oxaliplatin, and capecitabine (Bazarbashi et al., 2015) in combination with bevacizumab on a prospective clinical trial (clinicaltrial.gov: NCT01311050), were retrospectively reviewed for the location of the primary tumor. Right-sided tumors were defined from cecum to hepatic flexure. Left-sided tumors from splenic flexure to and including the rectum. Transverse colon patients were excluded. Patients who had multiple primaries were grouped with right-sided patients if at least one of primaries was right sided. Prognostic factors that might affect survival was recorded for right vs left sided primaries. Those include age, gender, performance status, KRAS status (NRAS was not tested as many of the paraffin blocks were exhausted), adjuvant chemotherapy, and the number of metastatic organs involved. Response rate (RR), progression-free survival (PFS) and overall survival (OS) was calculated for right sided and left sided primaries separately. As previously described, RR was assessed using response criteria in solid tumor (RECIST V1.1) (Eisenhauer et al., 2009). All patients received the combination of capecitabine, oxaliplatin, Irinotecan, and bevacizumab as per the doses described in the earlier publication of 5-8 cycles then maintained on capecitabine and bevacizumab. Univariate analysis was performed on the prognostic factors listed above.

*Ethical consideration*

The original study was approved by the institutional review board. All patients consented to the study. The retrospective review of sidedness was approved by the hospital ethical committee/Institutional review board. Patient confidentiality was maintained at all time. 

*Statistical analysis*

Patient characteristics were summarized using frequencies for categorical variables and medians with ranges for continuous variables. Further, categorical variables and continuous variables were compared using the Chi-squared test and Wilcoxon rank-sum test, respectively.

Survival probabilities were calculated using the Kaplan-Meier estimator with variance estimated using Greenwood’s formula(John P. Klein, 2005). Survival curves were compared using log-rank test.

OS was calculated from the date of registration till the date of last follow up or death. Death of any cause was considered as an event. PFS was calculated from the date of registration till the date of progression or death. Patients who were alive progression-free were censored at the date of last follow up. Disease progression and death were considered as events for this outcome.

Hazard ratios were calculated using Cox proportional hazard model after testing for proportionality assumption. In case of assumption, violation variable will be included as a time-dependent covariate.

## Results

**Figure 1:**
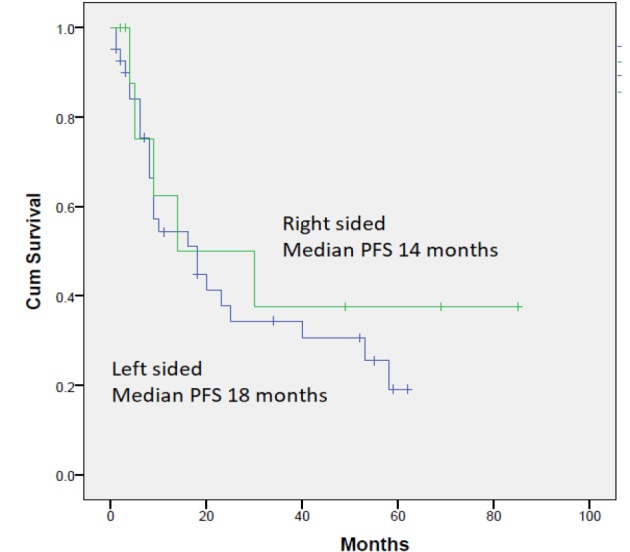
Kaplan-Meier Curve for Progression-free Survival in Patients with Metastatic Colorectal Cancer Treated with Triplet First-line Therapy from Right-sided vs Left sided Primaries. Median PFS 14 and 18 months for right sided (green line) and left sided (blue line) consecutively (Hazard ratio (HR) 0.72, 95% confidence interval (CI) 0.27-1.88, p=0.492)

**Figure 2.**
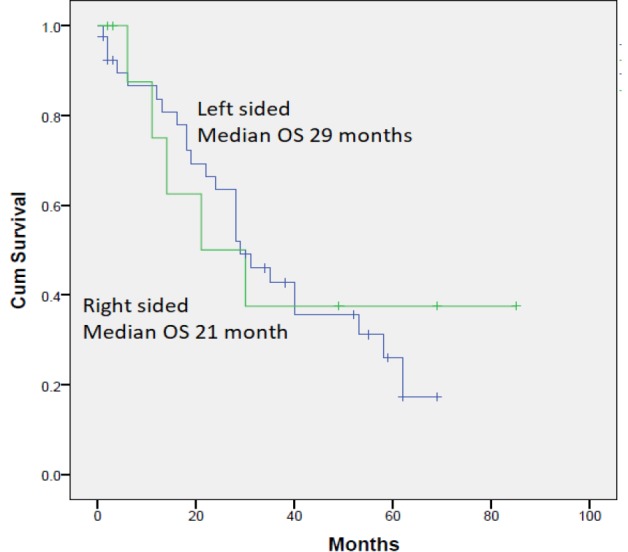
Kaplan-Meier Curve for Overall Survival in Patients with Metastatic Colorectal Cancer Treated with Triplet First-line Therapy from Right-sided vs Left sided Primaries. Median OS 21 and 29 months for right (green line) and left-sided (blue line) tumors consecutively (HR was 0.86, 95% CI 0.32-2.26, p=0.752)

Fifty-three patients were enrolled in the prospective phase I-II trial of the combination of irinotecan, oxaliplatin, capecitabine, and bevacizumab in advanced colorectal cancer. Out of those, 11 had right-sided tumors and 42 had left-sided tumors. The characteristics of each group are outlined in Table 1. Of note, patients with right-sided tumors had a younger age (the difference was statistically significant), and less incidence of liver metastasis. Other differences in patients’ characteristics were all not statistically significant likely because of small numbers. Median PFS was 14 and 18 months for right sided and left sided consecutively (Hazard ratio (HR) of 0.72, 95% confidence interval (CI) 0.27-1.88, p=0.492) (Figures 1). Median OS was 21 and 29 months for right and left-sided tumors consecutively (HR was 0.86, 95% CI 0.32-2.26, p=0.752) (Figures 2). Univariate analysis of the prognostic factors reported above is shown in Table 2. It showed the absence of liver metastasis as the only statistically significant factor in this group of patients with median OS in patients with liver metastasis vs those without liver metastasis of 22 months (95% CI 13.3-30.3) vs 58 months (95% CI 0.0-0.0), P= 0.004. 

## Discussion

The prognostic significance of sidedness for the primary tumor in metastatic colorectal cancer has been extensively studied in the past. Two large recent meta-analyses have established that metastatic colorectal cancer from right-sided primary has shorter survival than left-sided ones (Yahagi et al., 2016; Petrelli et al., 2017). In the meta-analysis presented by Yahagi and his colleagues, 15 studies were included with a total of 108,474 patients. In this meta-analysis, right-sided colon cancer had significantly worse overall survival than left-sided colon cancer with HR of 1.14, 95 % CI 1.06–1.22, p<0.01). Subgroup analyses demonstrated significant prognostic differences in western countries (HR=1.15, 95% CI 1.08–1.23, p<0.01), a nationwide database (HR=1.15, 95% CI 1.05–1.27, p=0.01), and a stage-adjusted analysis (HR=1.14, 95 % CI 1.05–1.24, p<0.01). The second meta-analysis published by Petrelli and colleagues, 66 studies were included with a total of 1,437,846 patients. In their report, left-sided colonic tumors were associated with a significantly reduced risk of death (HR, 0.82; 95%CI, 0.79-0.84; P < .001). This risk reduction was independent of stage, race, adjuvant chemotherapy, year of study, number of participants, and quality of included studies. It is important to note that both meta-analyses included patients of all stages. Non-of them looked at the aggressiveness of therapy (triplet vs doublet). 

**Table 1:** Patients’ Characteristics of Right Sided and Left Sided Tumors

Characteristic	Right-sided colon cancers (n=11)	Left-sided colon cancers (n=42)	P-value
Age (years)			
Median (range)	46 (24-55)	53.5 (32-74)	0.047
≤ 40 years	3 (27.3)	8 (19.0)	0.67
>40 years	8 (72.7)	34 (81.0)	
Gender			0.73
Female	6 (54.5)	19 (45.2)	
Male	5 (45.5)	23 (54.8)	
ECOG performance status			0.62
0 -1	9 (81.82)	37 (88.10)	
2	2 (18.18)	5 (11.90)	
Adjuvant chemotherapy			0.324
Yes	0 (0.00)	6 (14.29)	
No	11 (100.00)	36 (85.71)	
Resection of primary tumor			0.308
Yes	8 (72.73)	21 (50.00)	
No	3 (27.27)	21 (50.00)	
Liver metastases			0.478
Yes	6 (54.55)	29 (69.05)	
No	5 (45.45)	13 (30.95)	
Number of metastases			0.496
1	5 (45.45)	14 (33.33)	
2-4	6 (54.55)	28 (66.67)	
White blood cells (10^9/L)			0.708
<= 10.0	7 (65.64)	31 (73.81)	
>10.0	4 (36.36)	11 (26.19)	
Alkaline phosphatase			0.571
<=300	11 (100.00)	37 (88.10)	
>300	0 (0.00)	5 (11.90)	
Platelets (10^9/L)			0.173
<=400	5 (45.45)	29 (69.05)	
>400	6 (54.55)	13 (30.95)	
Hemoglobin (g/L)			0.173
<110	6 (54.55)	13 (30.95)	
>= 100	5 (45.45)	29 (69.05)	
KRAS gene mutation			1.000
Wild	3 (50.00)	12 (57.14)	
Mutant	3 (50.00)	9 (42.86)	

ECOG, Eastern cooperative oncology group

**Table 2:** Univariate Analysis of Different Prognostic Factors in the Patients Treated with Triplet Therapy

Item	PFS (mo)(95% CI)		OS (mo) ( 95% CI)	
Side		p-value		p-value
Right	14 (0.0-43.1)	0.492	21 (0.0-43.1)	0.752
Left	18 (5.9-30.0)		29 (20.2-37.8)	
Age				
≤ 40 years	20 (15.1-24.8)	0.747	40 (27.8-52.2)	0.487
> 40 years	14 (2.2-25.8)		28 (20.9-35.0)	
Gender				
Male	14 (0.0-28.5)	0.810	28 (27.2-38.7)	0.885
Female	18 ( 2.5-33.5)		29 (24.6-33.4)	
ECOG performance status				
0-1	18 (9.5-26.5)	0.696	30 ( 20.6-39.3)	0.999
2	8 (4.9-11.0)		28 (0.0-73.1)	
Liver metastases				
Yes	9 (0.75-17.2)	0.026	22 (13.3-30.3)	0.004
No	58 (0.0 -124.7)		58 (0.0-0.0 )	
Number of organs involved				
one	30 (4.8-55.2)	0.417	40 (28.8-51.2)	0.230
> 1	10 (0.6-19.4)		28 (22.4-33.6)	
White blood cell count				
≤ 10 K	16 (2.9-29.0)	0.997	31 (21.3-40.6)	0.446
> 10 K	18 (0.77-35.2)		19 (4.6-33.3)	
Platelets				
≤ 400 K	16 (3.3-28.7)	0.624	29 (20.0-37.9)	0.673
> 400	18 (0.0-38.9)		30 (14.8-45.2)	
Hemoglobin (mg/dl)				
≤ 11	20 (8.2-31.7)	0.431	30 (13.5-46.5)	0.651
> 11	16 1.4-30.5		29 (22.3-35.6)	
Alkaline phosphatase				
≤ 300	18 (9.6-26.3)	0.206	29 (19.7-38.3)	0.353
> 300	9 (3.9-14.0)		22 (20.2-33.8)	
KRAS gene mutation				
Wild	14 (3.1-24.8)	0.488	29 (18.1-39.8)	0.206
Mutant	8 (3.5-12.4 )		19 (7.6-30.3)	

PFS, progression free survival; OS, overall survival; ECOG, Eastern cooperative oncology group.

Whether increasing intensity of therapy in patients with metastatic disease, change the prognostic significance of sidedness is not clear. It appears that sidedness is prognostic in patients treated with fluoropyrimidine alone with HR for survival in both studies for right-sided vs left-sided tumor 1.5 (adjusted P = 0.0001) and 1.592 (95% CI 1.07–2.37, adjusted p= 0.022) respectively (O’Dwyer et al., 2001). Sidedness has also been of statistical significance in patients treated with modern doublet chemotherapy combined with targeted therapy. This has been clearly demonstrated in the CALGB/SWOG 80405 trial, the FIRE-3 trials, CRYSTAL trial and the meta-analysis published by ESMO (Tejpar et al., 2016; Alan P. Venook, 2017; Arnold et al., 2017). In the CALBG/SWOG 80405 trial, 1137 patients with wild-type K-ras were treated with FOLFOX or FOLFIRI with either Cetuximab or bevacizumab. Median overall survival for right-sided tumors was 19.4 months and for left-sided tumor was 34.2 months with HR for right-sided tumors of 1.56 (95% CI 1.32-1.84, P<0.0001). The difference was less marked for 252 patients with mutant K-ras treated prior to the trial amendment with median OS of 23.1 vs 30.3 months for right and left-sided tumors (HR 1.28, 95% CI 0.95-1.73, P not significant) (Alan P. Venook, 2017). In the CRYSTAL trial, with RAS wild-type metastatic colorectal cancer, 280 (76%) had left-sided tumors and 84 (23%) had right-sided tumors. Median survival for right-sided tumors treated with FOLFIRI plus cetuximab was 18.5 months vs 28.7 months for left-sided ones with HR of 1.93 (95% CI 1.24-2.99, p=0.003), while the median survival for right-sided tumors treated with FOLFIRI was 15 months vs 21.7 months for left-sided ones with HR of 1.35 (95% CI 0.93-1.97, p= 0.11) (Tejpar et al., 2016). In the final RAS wild-type population of FIRE-3 trial (n = 400), 306 patients had left-sided tumors (76.5%), and 88 had right-sided tumors (22%). Median survival for right-sided tumors treated with FOLFIRI plus cetuximab was 18.3 months vs 38.3 months for left-sided ones with HR of 2.84 ( 95% CI 1.86-4.33, p<0.001), while the median survival for right-sided tumors treated with FOLFIRI plus bevacizumab was 23 months vs 28 months for left-sided ones with HR of 1.48 (95% CI 1.02-2.16, p= 0.04) (Tejpar et al., 2016).

The only trial addressing the benefit of triplet therapy in right-sided tumors vs left-sided tumors was the TRIBE trial (Cremolini et al., 2015). In this trial, 508 patients with untreated metastatic colorectal cancer were randomized to receive either FOLFIRI plus bevacizumab or FOLFOXIRI plus bevacizumab. In a subgroup analysis, right-sided tumors had more progression-free survival benefit with HR of 0.66 for the FOLFIXIRI arm than left-sided tumors with HR of 0.82, p=0.20.

Our study is the first one to our knowledge looking at the prognostic significance of sidedness of primary tumor in patients with metastatic colorectal cancer treated primarily with first line triplets’ therapy. Despite that, the difference in median overall survival between right sided and left sided tumors were higher in left-sided tumors, the hazard ration for both PFS and OS was in favor of left-sided tumors with no statistical significance likely because of small sample size. 

Of note, it is worth mentioning that triplets therapy in patients with metastatic colon cancer have considerable toxicity and is only feasible in patients with good performance status. 

The study has its own limitations including a small sample size, single institution and the presence of imbalance in the patients’ characteristics between right and left sided groups.

In conclusion, our study demonstrated no significant difference in survival in patients with right versus left primary metastatic CRC treated with triple first-line chemotherapy and bevacizumab. This finding is hypothesis generating and need to be confirmed. A pooled analysis of all data published of triplets’ first-line chemotherapy in metastatic CRC is warranted. 

*Disclosure and acknowlegment*

This study has not received any financial and/or material support that was received for the research or the creation of the work. Also, none of the authors have any relationships (personal, academic or financial relationships that could influence their actions) or financial involvement with an organization or entity with a financial interest in or financial conflict with the subject matter or materials discussed in the manuscript. No writing assistance has been used in the creation of the manuscript.

The original study was approved by the institutional review board. All patients consented to the study. The retrospective review of sidedness was approved by the hospital ethical committee/Institutional review board. Patient confidentiality was maintained at all time. 
